# Psychological impacts of intervention to improve a therapeutic garden for older adults with dementia: a case study conducted at a care facility

**DOI:** 10.3389/fpsyt.2023.1183934

**Published:** 2023-05-10

**Authors:** Chiara Meneghetti, Veronica Murroni, Erika Borella, Andrea Melendugno, Elena Carbone, Giulia Goldin, Raffaele Cavalli, Andrea Basso, Francesca Pazzaglia

**Affiliations:** ^1^Department of General Psychology, University of Padova, Padova, Italy; ^2^Inter-University Research Center in Environmental Psychology (CIRPA), Roma, Italy; ^3^Casa Madre Teresa di Calcutta (O. P. S. A.), Padova, Italy; ^4^Department of Land Environment Agriculture and Forestry, University of Padova, Padova, Italy; ^5^Giotto Social Cooperative Padova, Padova, Italy

**Keywords:** exposure to nature, therapeutic garden, dementia, behavior mapping, intervention

## Abstract

**Introduction:**

Exposure to nature is known to support psychological wellbeing, and can support People with Dementia (PwD). Here we describe a case study conducted at a care facility for PwD to examine the effect of their exposure to nature after intervention to renovate an existing Therapeutic Garden (TG). Changes in frequency of attendance and behavior in the TG were examined. A single case was also considered to assess individual benefits.

**Materials and methods:**

Twenty-one PwD participated in the study. Their behavior in the TG was observed for 4 weeks before and after the intervention (using behavioral mapping), and measures of individual characteristics (general cognitive functioning, behavioral/neuropsychiatric symptoms, depression, and quality of life) were administered.

**Results:**

Ten of the 21 PwD visited the TG more often after the intervention, their social behaviors (e.g., talking to others) increased, and their active isolated behavior in the garden (e.g., smelling, touching flowers) tended to increase. The increase in social behavior related to less severe baseline depressive symptoms. Passive isolated behaviors related to more impaired baseline cognitive functioning. The case of Mrs. A extended the findings for the whole sample: although her dementia symptoms (apathy, motor disturbances) worsened, she visited the TG more often after the intervention, her social exchanges and active isolated actions increased, and her agitation and wandering decreased.

**Discussion:**

These results support the benefits of exposure to nature for PwD, and underscore the importance of considering users’ profiles to optimize their use of a TG.

## 1. Introduction

The positive effects of nature on humans are well established. The main theories explaining why interaction with nature is beneficial to humans are Stress Reduction Theory [SRT; (1, 2)], and Attention Restoration Theory [ART; (3, 4)]. These theories provide the frames of reference for empirical evidence of the benefits of exposure to nature in reducing stress and promoting positive affective and health states [(5, 6) for a review]. Other evidence suggests that exposure to nature produce benefits in cognition [memory and attention; (7, 8)]. According to ART, there are four key components that characterize a restorative environment: fascination (the property of the environment to hold our attention with no voluntary effort on our part); extent (the opportunity to feel immersed in the environment); being away (establishing a distance between us and our everyday routine); and compatibility (with our own inclinations). It is hard to say which key environmental features are most relevant for humans interacting with nature. The complexity increases when individuals’ personal characteristics are considered, especially for those with behavioral and cognitive disorders like people with dementia (PwD).

Dementia is a neurocognitive disorder characterized by a gradual decline in cognitive functions (e.g., memory, orientation) and deterioration in emotional, social, and behavioral control that affect individuals’ day-to-day autonomy (DSM-V). With a greater life expectancy comes an increase in the incidence of dementia—and its related sub-types [(9); e.g., (10)]—among older adults. Therefore, to consider the potential benefits of the interaction with nature is a resource for PwD.

Favoring contact with nature in a protected and stimulating environment is known to be important for PwD, helping them to manage and compensate for their dementia symptoms, and favoring their psychological wellbeing (11–14). Therapeutic gardens (TGs) installed at dementia care centers are designed to emphasize this curative potential. TGs offer PwD a garden setting inside or outside the care center, where they can simply sit or walk, or engage in gardening or other activities (15).

A recent systematic review (16) on TGs for dementia (*N* = 16 studies) summarizes and clarifies their features and benefits. Visiting TGs where PwD can sit or stand, interact freely with natural elements, or walk, is related to a reduction in behavioral symptoms, with less aggressiveness and agitation (17–20). Exposure to TGs also seems to improve mood [(21)—less depression; (22)], quality of life (17, 22), cognitive functioning (20), engagement in the surroundings (23), and social interaction [comments about the garden, and response to caregivers; (24)]. Only a few of the studies reviewed mentioned the types of plants involved when examining the benefits of TGs. When Pedrinolla et al. (20) examined how PwD interacted freely with an indoor TG, an experimental group that wandered around the TG, touching plants and brightly-colored flowers (e.g., *Ficus benjamina*, *Croton variegatum*), and smelling aromatic herbs (e.g., *Rosmarinus officinalis*), was compared with a control group that did not visit the TG. The experimental group showed signs of greater psychological wellbeing than in the control group, with less behavioral disturbance and stress, and showed benefits in cognitive functioning. In another study, Collins et al. (17) observed PwD interacting with plants with features that promoted the use of touch, taste, sight, smell (i.e., *Coriandrum sativum*, *Lactuca sativa “Simpson Elite”*) in an indoor and an outdoor garden. The results showed an overall reduction in agitation and improvement in quality of life in both gardens, especially the one outdoors. These two studies show that TGs promote interaction with nature in PwD, thereby reducing their behavioral symptoms. They also suggest that attention should be paid to the choice of vegetation and type of garden.

Some guidelines on TGs suggest which natural elements and vegetation can facilitate interaction with nature for PwD (25). A multi-sensory experience should be promoted by choosing plants and flowers that can be touched or have soothing scents to promote olfactory, visual, tactile stimulation, but also to attract birds and butterflies (26). Trees should be chosen to provide shade, color, seasonal variety, and the sound of leaves rustling in the wind (18). Some authors recommend arranging TGs and choosing plants with the specific features of PwD in mind [i.e., dementia severity profile; (27, 28)]. Such TGs should be designed with plants or elements that can sustain psychological wellbeing and quality of life by promoting a sense of calm, immersion in the setting, and psycho-physical balance, and by promoting motor activity and social exchanges between users (29).

Most studies on the topic have focused on the effects of exposing PwD to TGs in terms of their dementia symptoms and psychological aspects (mood, wellbeing) (16), but there is a paucity of evidence regarding specific interventions in gardens (26) and in relation to their individual dementia profiles. It would be interesting to see whether ameliorating a TG, by adding various plant species (flowers and shrubs) that could maximize its therapeutic and stimulating potential, could add to its beneficial effects on PwD.

The main aim of the present paper is to examine whether and to what extent intervention to improve the greenery maximizes the beneficial and stimulating effects of a TG for PwD, influencing the frequency of their visits and their behavior in the garden. This was examined considering a sample of PwD at the care facility where the TG intervention was completed, mapping their behavior before and after the intervention. The relationship between their garden visiting and behavior patterns with their individual characteristics (cognitive functioning, mood, behavioral disturbances and quality of life) was also assessed. To qualitatively corroborate the beneficial effects of the modified TG, the case of Mrs. A is presented. The case was selected to analyze the positive changes in her behavior after the intervention, despite her worsening dementia.

## 2. Case study

### 2.1. Participants

The sample was recruited from a care facility in Northeast Italy. Eligibility was restricted to individuals with: (i) a diagnosis of major neurocognitive disorder [of any etiological subtype) made by the patients’ reference clinical center for cognitive decay (DSM-5; (30)]; (ii) global functioning score (using the Montreal Cognitive Assessment) compatible with mild-to-moderate cognitive impairment, and (iii) autonomous locomotion, or supported by mobility aids.

Twenty-one participants were eligible (9 females; 38% of the sample; mean age: 78,76; SD = 8.53; mean years of education: 6.95 SD = 2.75; 8 [38%] lived in and 13 [62%] attended for daycare).^1^ The MOCA scores (*M* = 11.77; SD = 5.52) confirmed mild-to-moderate cognitive impairment [converting MOCA scores to Mini-Mental State Exam (31) scores they are included in the range 18–13; see (32)].

### 2.2. Materials

#### 2.2.1. Behavioral mapping

Behavioral mapping [inspired by (33)]. This assesses: (i) the frequency of visits to the garden and locations where PwD stopped and (ii) their behaviors, classified as passive isolated, active isolated, social, aggressive, agitated, conscious movement, and disoriented movement (wandering). Supplementary Table S1 shows the list of specific behaviors for each category.

#### 2.2.2. General cognitive functioning

*Montreal Cognitive Assessment* [MoCA; (34)]. This consists of 30 items that test visuospatial abilities, executive functions, language, delayed recall, attention, and temporal and spatial orientation. The dependent variable was the sum of the scores (max 30), corrected for age and education. A score below the cut-off of 17 indicates cognitive impairment (35).

*Alzheimer’s Disease Assessment Scale-Cognitive subscale* [ADAS-Cog; (36)]. This consists of 11 tasks assessing orientation, memory, language, praxis, attention, and other cognitive abilities. The variable was the sum of the scores (max = 70), with higher scores indicating a more impaired cognitive functioning.

#### 2.2.3. Mood

*Cornell for Depression in Dementia scale* [CDDS; (37)]. This consists of 19 items assessing signs and symptoms of major depression in individuals with dementia. Each item is rated for severity on a scale from 0 (absent) to 2 (severe). The variable was the sum of the scores (max = 38), with higher scores indicating more severe depressive symptoms (scores between 1 and 10 indicate probable major depression, and scores above 11 major depression).

#### 2.2.4. Behavior

*Neuropsychiatric Inventory* [NPI; (38)]. This assesses 12 behavioral disturbances in dementia patients (delirium, hallucinations, agitation, dysphoria, anxiety, euphoria, apathy, disinhibition, irritability, motor disturbances, sleep disturbances, and food issues). For each disturbance, the caregiver’s emotional and psychological distress can also be measured. The variables were: (i) the sum of the frequency × severity scores on each symptom and (ii) the sum of the caregiver’s distress scores on each symptom. Higher scores (max = 144) indicate more frequent and more severe disturbances, and more severe caregiver distress.

#### 2.2.5. Quality of life

*Quality of Life-Alzheimer’s Disease scale* [QoL-AD; (39)]. This consists of 13 items assessing subjective components of quality of life (e.g., perceived quality of life and psychological wellbeing), and objective components (e.g., behavioral competence and environment), rated by caregivers on a 4-point scale from 1 (poor) to 4 (excellent). The variable was the sum of all the items (max = 52), where higher scores indicate a better quality of life.

### 2.3. Greenery intervention

**Figure 1:**
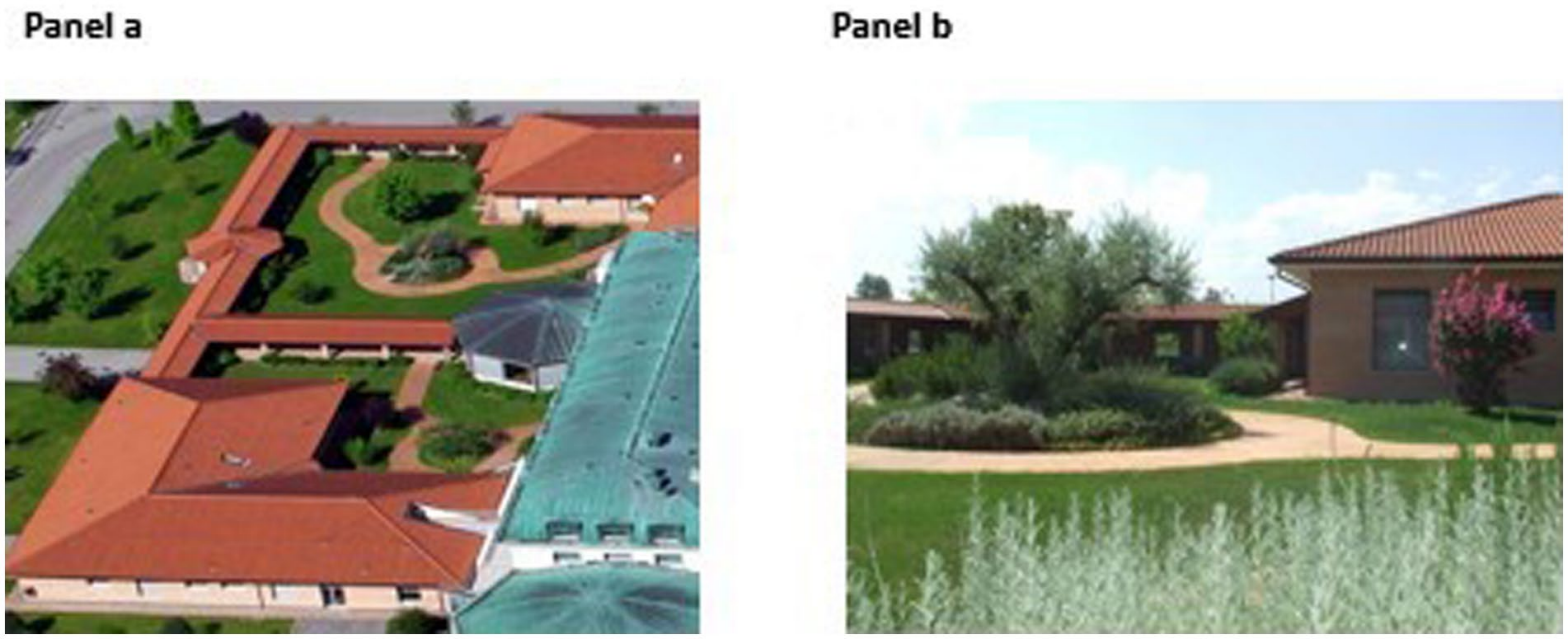
The therapeutic garden: aerial view (Panel a) and observer’s view (Panel b) before the intervention.

**Figure 2:**
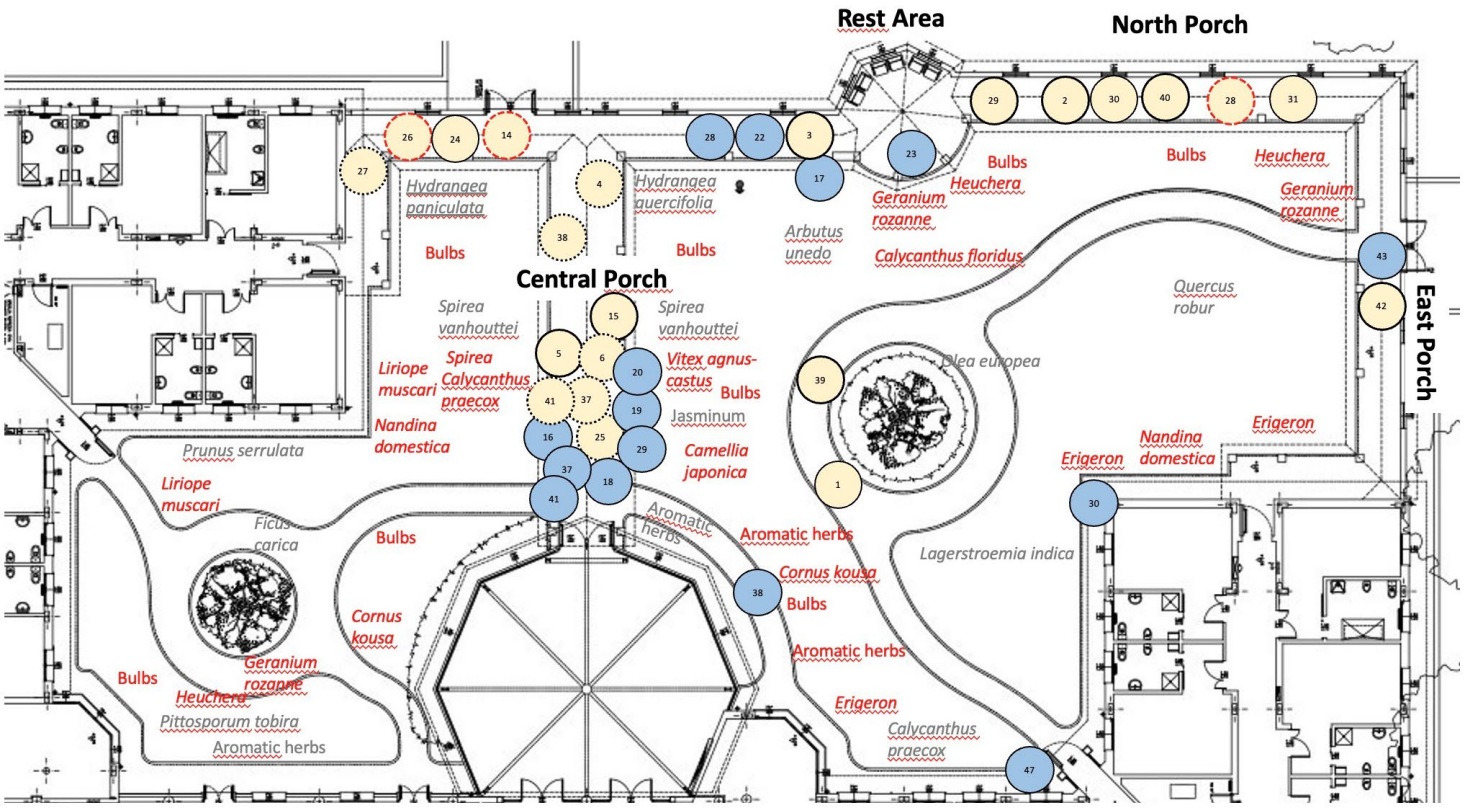
The layout of one part of the TG. TG layout showing the plants before the intervention in gray, those added with the intervention in red. It is also reported the behavioral mapping of Mrs A: the numbers indicate the observation times (blue circles for times 1–48 pre-intervention; pink circles for times 1–48 post-intervention). Post-intervention locations where Mrs. A engaged in active isolated behaviors: 2, 3, 5, 14, 15, 26, 28, 29, 39, 40, and 42 (circles with continuous lines). Locations where Mrs. A engaged in social behaviors: 4, 6, 14, 25, 26, 27, 28, 37, 38, and 41 (circles with dotted lines). At locations 14, 26, and 28 (circles with red dotted lines), she engaged in both active isolated and social behaviors. At locations 1, 24, 30, 31, she started wandering.

The TG covers an area of 2,500 m^2^ at the care center for PwD. It is divided into three similarly-organized parts. Figure 1 shows an aerial view and an observer’s view of one part prior to the intervention; Figure 2 shows the layout of one part of the TG. The TG was originally designed (in 2005) by gardening experts according guidelines (25). There are 4 medium-to-large trees (*Quercus robur, Ficus carica, Prunus serrulata, and Olea europaea*), about 40 small-to medium-sized shrubs (e.g., *Spirea vanhouttei, Lagerstroemia indica*), and aromatic herbs (about 20, *Rosmarinus officinalis* and *Salvia officinalis*) to provide flowers and colorful foliage through the seasons. The intervention completed in 2021 (October) was based on guidelines [e.g., (25)], and on unanimous request coming from staff and family members (assessed with an *ad hoc* questionnaire; *N* = 18; May 2021) to increase the vegetation. More small-to medium-sized shrubs were added to provide seasonal flowers and colorful foliage. The species-planted near walkways and rest areas-are shown in Figure 2 (in red). There are about 450 late winter and spring flowering bulbs with a variety of colors and smells (e.g., *Crocus chrysanthus* and variegated tulips). There are about 200 shrubs that flower at different times of year (e.g., *Vitex agnus-castus*, *Nandina domestica*, *Cornus kousa*, *Calycanthus praecox*), and about 20 aromatic herbs (*Rosmarinus officinalis* and *Salvia officinalis*).

### 2.4. Procedure

**Figure 3:**
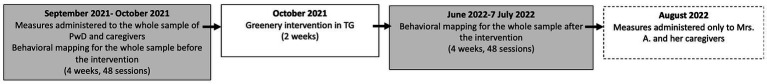
Timeline of the intervention. The rectangle with the dotted lines only concerns Mrs. A (case study).

The study began in September 2021 and ended in July 2022 (see Figure 3 for the timeline) in compliance with COVID-19 measures during both periods with temperatures suitable for staying outside. In September tests and questionnaires were administered to PwD (MoCA, ADAS-Cog) and their caregivers (CDDS, NPI, QoL-AD). From September to October 2021 (14–19\u00B0C°), the pre-intervention behavioral mapping of participants was performed for 4 weeks. The TG intervention was completed in October (2021; taking 2 weeks). From June to July 2022 (19–14\u00B0C°), post-intervention behavioral mapping was performed for 4 weeks for the maximum seasonal flowering. The behavioral mapping involved recording behaviors twice a day (once in the morning 10–12 h., once in the afternoon 13–15 h.) at times when PwD were not engaged in other activities and were free to access the garden if they wished. The mapping lasted 4 weeks, with 6 behavior recording times in each session (at the start, then every 10 min for 50 min in all) for a total of (2x4x6=) 48 behavior recordings before and 48 after the intervention. The data were collected by two psychologists at a time (one was the first observer) and for the analyses we considered the first observer’s data (as they correlated closely with the other psychologist’s both pre-and post-intervention, Rho_s_ = 0.99 p_s_ ≤ 0.001).

### 2.5. Results

Frequency of visits to the TG and behaviors seen in PwD before and after the intervention. With a preliminary analysis on the number of visits to the garden we detected three ranks: negative (3 post<pre), positive (10 post>pre), or equal (8 post = pre; Wilcoxon test z = 2.17 *p* = 0.030). Ten people with PwD significantly increased the number of visits to the garden after the intervention (pre: M = 6.00, SD = 4.32: post: M = 13.70, SD = 8.59; min +1 max +27; Wilcoxon test: z = 2.81 *p* = 0.005). No differences in demographic variables (age, education) and baseline measures emerged between the PwD group who visited the garden more (*N* = 10) and the group who did not (*N* = 11) (Kruskal Wallis, z = 0.64–1.23 p_s_ > 0.22; with the exception of QoL-AD scores, z = 1.95 *p* = 0.052; see Supplementary Table S2).

From pre-to post-intervention, the 10 PwD who visited the garden increased their social interactions (Wilcoxon test; *z* = 2.09 *p* = 0.04; pre: *M* = 4.30, SD = 3.20; post: *M* = 9.70, SD = 5.38) and showed a tendency to increase their active isolated actions in the garden (Wilcoxon test; z = 1.87 *p* = 0.06; pre: *M* = 1.10 SD = 1.85; post: *M* = 4.40, SD = 6.85). See Supplementary Table S3.

Associations between baseline measures and changes in the frequency of visits and behaviors exhibited by PwD in the garden. We expected a less impaired neuropsychological profile to be associated with a greater benefit of the intervention. One-tailed Spearman correlations between the gains in the number of visits to the garden and the frequency of certain actions (numbers post-intervention minus numbers pre-intervention) and the baseline scores for cognitive functioning, mood, behavioral disturbances and quality of life (i.e., pre-intervention scores in the respective measures for the 10 PwD who visited the TG) showed that: the gain in social interactions was inversely related to the baseline CDDS score (Rho = −0.61 *p* = 0.031), i.e., PwD with less severe depressive symptoms at the baseline engaged in more social interactions after the intervention on the TG; and the gain in passive isolated behaviors (that increased slightly post-intervention) was directly associated with baseline ADAS-Cog (Rho = 0.62 *p* = 0.027), i.e., it was the PwD with a worse cognitive functioning (higher scores) at the baseline whose passive isolated behaviors increased.

## 3. Case description

### 3.1. Mrs. A

Mrs. A was one of the PwD whose visits to the TG increased from pre-to post-intervention (September 2021 to July 2022) despite a worsening of her behavioral symptoms. The changes qualitatively perceived by the staff prompted a new assessment of her general cognitive functioning, mood, behavior and quality of life (in August 2022; See Figure 3).

Mrs. A is a 70-year-old with a diagnosis of Alzheimer’s disease. She once worked as a nurse. She is widowed, and has three children. She loved cooking and gardening. Before entering the center, she often tended to wander, and would get lost. On arrival in 2019 (3 years before our study) her cognitive impairment was moderate–severe [Mini-Mental State Exam–MMSE: 11.90/30; (31)], associated with agitation and wandering. By 2021 she had stopped speaking and seeking contact and interaction with others. She moves autonomously indoors and was able to go out into the garden unassisted.

### 3.2. Procedure

Same as in the case study, but the measures administered at pre-intervention (MoCA, ADAS-Cog, CDDS, NPI, QoL-AD; see Figure 3) were administered again at post-intervention.

### 3.3. Results

In the 12 months (September 2021 to August 2022), Mrs. A’s cognitive functioning level worsened, making it impossible to administer the MoCA or ADAS-Cog at post-test (MoCA pre = 10.4, post = NA; ADAS-Cog pre = 53.3, post = NA). The frequency and severity of her behavioral symptoms worsened (NPI pre- = 12, post = 20), with more severe apathy, motor disturbances, and food issues, her depression became more severe (CDDS pre = 0, post = 4), and her quality of life deteriorated (QoL-AD pre = 30, post = 24).

Despite her increasingly severe dementia profile, she increased the number of visits after the TG changed (pre: 15, post: 22). She engaged in more active isolated behaviors (pre: 0, post: 14), and social behaviors (pre: 3, post: 15); she no longer became agitated (pre: 8, post: 0), and her wandering episodes decreased (pre: 13, post: 7). She did not engage in any of the other behaviors (passive, aggressive, moving alone, moving with others; see Supplementary Table S4 for the complete scores). Her specific active isolated and social isolated behaviors after the intervention are listed in Table 1, and the places where she exhibited these behaviors after the intervention are shown in Figure 2 (pink circles). Supplementary Table S5 shows all the behaviors she exhibited pre-and post-intervention in chronological order (behavior observation times 1–48).

**TABLE 1:** The case of Mrs. A–specific active isolated and social behaviors after the intervention.

	Behaviors and frequency	Behavior observation times (1–48)*
Active isolated (*N* total: 14)	Sitting in the garden, observing natural elements and other people (* N * = 2)	2, 26
	Paying attention to outdoor spaces without prompting (* N * = 6)	2, 3, 5, 14, 28, 29
	Observing, smelling, touching flowers without prompting (* N * = 4)	3, 39, 40, 42
	Noticing the presence of a bird in the garden (* N * = 1)	26
	Eating something offered by staff (* N * = 1)	15
Social (N total: 15)	Interacting with others through non-verbal communication, e.g., smile, look in eyes (* N * = 5)	4, 6, 25, 37, 41
	Giving flowers to staff (* N * = 1)	6
	Observing, smelling, touching flowers with other people (* N * = 1)	6
	Greeting gardeners (* N * = 1)	14
	Joining another person in the garden (* N * = 7)	25, 26, 27, 28, 37, 38, 41

The locations where these behaviors occurred are shown in Figure 2.

## 4. Discussion

The results of the study on the whole sample and a single case show the benefits of intervention to improve an existing TG at a dementia care center. The intervention involved adding various types of medium-sized plants (flowers and shrubs) to provide more color (with branches and flowers) and smells in all seasons.

The results on the whole sample showed that after the intervention, there was an increase in the number of visits made by around half of the PwD (10 out of 21; 47.62%). These 10 PwD already visited the garden before the intervention, but did so much more afterward (doubling their visits on average, albeit with a marked individual variability, from +1 to +27). The PwD who visited the TG more often after the intervention increased their social behaviors (e.g., talking more to other patients, commenting aloud on something in the garden) and their active isolated behaviors (e.g., observing, smelling, touching plants) tended to increase. These findings are in line with other reports of beneficial effects of TGs on PwD (16), with specific benefits detected in individuals’ engagement and interaction (23, 24), staying alone, interacting with elements of the garden, and communicating with others. The novelty of our study lies in that these behavioral improvements were obtained basically by improving the variety of plant species in the garden [not by means of specific activities; (16)].

The other novel result here concerns the relationship between behavioral gains (more behaviors after the intervention) and baseline individual characteristics (cognitive functioning, mood, behavioral disturbances, and quality of life). The correlations (for the 10 PwD visiting the garden more after the intervention) showed that the number of social behaviors increased after the intervention on the garden in individuals with less severe depressive symptoms at baseline (before the intervention), while passive isolated behaviors (which changed only slightly from pre-to post-intervention) were related to a worse cognitive functioning at baseline. These results suggest that lower depressive symptoms might have favored the expression of positive behaviors involving interaction with others in the garden after improving the greenery in a TG. Depressive symptoms usually interfere with the cognitive functioning and behavior of PwD (40), and our results indicate that less severe depressive symptoms in PwD favored the expression of positive (social) behaviors after the greenery in a TG was improved.

At the same time, we found a more impaired cognitive profile, and weaker available cognitive resources associated with more isolated behaviors in the garden (like sitting without showing any interest, snoozing). This result suggests that for PwD with a worse cognitive profile changing the greenery in a TG is not enough to increase their positive behaviors. A more accurate choice of natural elements and/or structured outdoor activities [such as horticulture; e.g. (16)] in relation to specific profile of PwD is an issue that warrants further investigation.

The analysis of the single case of Mrs. A qualitatively corroborates the benefits of intervention to improve a TG. Although her dementia symptoms worsened (in terms of cognitive functioning, depression, behavioral symptoms and quality of life) in the 12 months elapsing between before and after the intervention, her visits to the garden increased, and so did her active isolated behaviors (such as observing elements, noticing a bird in the garden) and social behaviors (interacting with others, giving flowers to the staff). Her wandering decreased and her episodes of agitated behavior in the garden disappeared. Gardens can have a calming effect on the behavioral symptoms of dementia [such as wandering and agitation; (20, 22)]. It should be noted that Mrs. A had a passion for gardening before she developed dementia, and this may be part of the reason why she spends time in the garden nowadays and interacts with its elements, increasing her functional behaviors despite her worsening clinical profile. This underscores the importance of the person-centered approach to quality-improving interventions, which in the present case involved making changes to the greenery in a TG.

Despite the promising results, this study has several limitations. Although the intervention to improve the TG produced benefits in 10 (out of 21) PwD, the other 11 never visited the TG, or did so less after the intervention. Although the two groups had similar profiles in terms of cognitive functioning, mood, behavioral disturbances (albeit with slight differences in quality of life) the sample size was small and prevented us from clearly depicting the effects of the intervention and the role of individual characteristics. The number of visits and behaviors might also be influenced by pre-and post-assessments being made in two different seasonal periods, though outdoor temperatures were pleasant in both (favoring visits in the garden), and the post-intervention coincided with the greatest seasonal flowering and color. Our findings support the conviction that individual characteristics would explain the benefits of interventions for PwD, as only some of our sample changed in their habits regarding the garden after its improvement. This issue needs to be approached in future studies on larger samples, and over longer times, considering the season (for flowering), and analyzing the role of individual characteristics more closely. It would also be worth examining the impact of exposure to nature on the well-known interplay between neurobiological changes and dementia symptoms [e.g., (41)], and whether interventions can counteract dementia symptoms at this level too.

## 5. Conclusion

This study offers a contribution on the positive effect of improvement interventions based on adding plant species in existing TGs for PwD. Such interventions seem to be beneficial in increasing the frequency of their visits and their functional behaviors, especially their social behaviors. This seems particularly evident in PwD with less severe depressive symptoms, assessed before the intervention. This line of research deserves to be furthered to identify customized TG design criteria, construction, and upgrading of TGs for people with dementia considering individual profiles to sustain their psychological wellbeing.

## Data availability statement

The raw data supporting the conclusions of this article will be made available by the authors, without undue reservation.

## Ethics statement

The study was approved by the Ethical Committee of University of Padova (Univoc number: B0ED79C242237AB75E1452E090F3880A; number of protocol: 4275). All participants were informed about the purposes of the study and gave their written informed consent in accordance with the Declaration of Helsinki (World Medical Association, 2013). The patients/participants provided their written informed consent to participate in this study.

## Author contributions

CM, VM, EB, AM, RC, AB, and FP contributed to the design, the implementation of research, the analysis of the results, and the writing of the manuscript. EC and GG contributed to data collection, the analysis of the results, and the writing of the manuscript. All authors contributed to the article and approved the submitted version.

## Funding

This research received specific grants from University Padova Project, Uni-Impresa (2019): “VERde e BENessere nell’Alzheimer (VER.BEN.A). Verso un modello di giardino terapeutico centrato sull’interazione luogo-persona” [Green and wellbeing in Alzheimer (Ver.Ben.a). Toward a model of therapeutic garden centered on the place-person interaction].

## Conflict of interest

The authors declare that the research was conducted in the absence of any commercial or financial relationships that could be construed as a potential conflict of interest.

## Publisher’s note

All claims expressed in this article are solely those of the authors and do not necessarily represent those of their affiliated organizations, or those of the publisher, the editors and the reviewers. Any product that may be evaluated in this article, or claim that may be made by its manufacturer, is not guaranteed or endorsed by the publisher.

## Footnotes

^1^The etiology for the 21 PwD was: 4 with Alzheimer’s disease; 4 with vascular dementia; 5 with mixed dementia; and 8 with other types of dementia.
