# Supplementary material for: Psychological impacts of intervention to improve a therapeutic garden for older adults with dementia: a case study conducted at a care facility
Source: Front Psychiatry. 2023 May 10;14:1183934. doi: 10.3389/fpsyt.2023.1183934 (PMC10206005; doi:10.3389/fpsyt.2023.1183934)
Supplement: Supplementary file 1 [file Data_Sheet_1.PDF]

Psychological impacts of intervention to improve a therapeutic garden for older adults with dementia: A case study conducted at a care facility

Supplementary material

Table S1. The behaviors mapped.

Category	Behaviors
Passive isolated	Sitting in the garden and snoozing; Sitting in the garden without showing any interest in the garden and its elements; Sitting in the garden in a confusional state; Behaviors focused on the self.
Active isolated	Sitting in the garden and observing natural elements or people; Observing, smelling or touching flowers, plants or trees without prompting; Commenting aloud on what the PwD sees, touches or smells; Noticing the presence of animals; Paying attention to outdoor spaces without prompting; Other behaviors (reading, writing, listening to music, smoking, etc.).
Social	Talking to other guests, staff members or relatives in the garden; Greeting other guests, staff members or relatives in the garden; Inviting other guests, staff members or relatives to do something together in the garden; Commenting on the garden and its elements with others; Observing, smelling, touching flowers with others.
Aggressive	Verbal aggression towards other guests, staff members or relatives; Physical aggression towards other guests, staff members or relatives.
Agitated	Laughing awkwardly, crying, shouting; hiding things in the garden; repetitive behaviors; complaining about physical pain, discomfort or state of mind; Sexual behavior.
Conscious movement	Going deliberately to a certain part of the garden (alone or with someone).
Disoriented movement	Walking, wandering aimlessly

Table S2. PwD (10) whose visits increased versus those who did not visit the garden (or their visits decreased) (11), by demographic variables and baseline measures

	**PwD who visited the garden more (N =10)**	**PwD who visited the garden less or not at all (N=11)**
Age	79.90 (8.99)	77.73 (8.37)
Education (years of schooling)	6.30 (2.63)	7.54 (2.84)
MoCA	13.50 (5.14)	10.21 (5.61)
ADAS-cog	42.72 (17.23)	52.24 (20.11)
NPI	16.00 (10.38)	21.45 (16.63)
CDDS	5.80 (4.42)	4.73 (4.45)
QoL-AD*	31.10 (3.76)	27.45 (4.30)

Note. MoCA: Montreal Cognitive Assessment: ADAS-Cog: Alzheimer’s Disease Assessment Scale - Cognitive subscale; NPI: Neuropsychiatric Inventory; CDDS: Cornell for Depression in Dementia Scale; QoL-AD: Quality of Life - Alzheimer's Disease scale. * Kruskal Wallis: z = 1.95 p =.052: tendency of higher scores for the group that visited the garden than for the group that did not.

Table S3. Means and standard deviations of the number of visits and behaviors assessed using behavioral mapping before and after the intervention.

Behaviors	Pre-intervention	Post-intervention
Passive isolated	0.10 (0.32)	1.00 (3.16)
Active isolated	1.80 (1.85)	4.40 (6.85)
Social	4.30 (3.20)	9.70 (5.38)
Aggressive	0.00 (0.00)	0.10 (0.32)
Agitated	0.80 (2.53)	2.40 (7.59)
Conscious movement	0.40 (1.04)	0.36 (0.66)
Disoriented movement	1.30 (4.11)	0.70 (2.21)

Table S4. Mrs. A.'s scores on the measures of interest and her behavioral mapping in the garden before and after the intervention.

	**Pre-intervention**	**Post-intervention**
Measures (score)		
MoCA	10.40	NA
ADAS-cog	53.30	NA
NPI	12	20
CDDS	0	4
QoL-AD	30	24
Behavioral mapping (frequency)		
Visits to the garden	15	22
Passive isolated	0	0
Active isolated	0	14
Social	3	15
Aggressive	0	0
Agitated	8	0
Conscious movement	0	0
Disoriented movement	13	7

Note. MoCA: Montreal Cognitive Assessment: ADAS-Cog: Alzheimer's Disease Assessment Scale - Cognitive subscale; NPI: Neuropsychiatric Inventory; CDDS: Cornell for Depression in Dementia Scale; QoL-AD: Quality of Life - Alzheimer's Disease scale.

Table S5. Behavioral mapping of Mrs. A. before and after the intervention by observation time (1-48). and behaviors recorded (1. Passive isolated. 2. Active isolated. 3. Social. 4. Aggressive. 5. Agitated. 6. Conscious movement. 7. Disoriented movement). including some specific behaviors noted.

	**Behaviors**	
Behavior observation times 1-48	**Pre-intervention**	**Post-intervention**
1		7
2		1: Sitting in the garden and observing natural elements or people;<br>2: Paying attention to outdoor spaces without prompting
3		2: Paying attention to outdoor spaces without prompting;<br>2: Observing. smelling or touching flowers. plants or trees without prompting
4		3: Interacting with others through non-verbal communications
5		2: Paying attention to outdoor spaces without prompting
6		3: Giving flowers to staff members;<br>6: Observing. smelling or touching flowers with other people;<br>3: Interacting with others through non-verbal communications
**7**		
8		
9		
10		
11		
12		
13		
14		2: Paying attention to outdoor spaces without prompting;<br>3: Greeting the gardeners;
15		2: Eating something offered by staff taking a break in the garden;<br>7
16	3: Greeting the gardeners;<br>5: Repetitive behaviors;<br>7	
17	5: Repetitive behaviors;<br>7	
18	7	
19	5: Repetitive behaviors;<br>7	
20	5: Repetitive behaviors;	
21		
22	5: Repetitive behaviors;	

	7	
23	5: Repetitive behaviors;<br>3: Interacting with others through non-verbal communications	
24		7
25		3: Interacting with others through non-verbal communications;<br>3: Joining another person in the garden
26		1: Sitting in the garden observing natural elements or other people;<br>2: Noticing the presence of a bird in the garden;<br>3: Joining another person in the garden
27		3: Joining another person in the garden
28	3: Greeting the gardeners;<br>7	3: Joining another person in the garden;<br>2: Paying attention to outdoor spaces without prompting
29	7	2: Paying attention to outdoor spaces without prompting
30	7	7
31		7
32		
33		
34		
35		
36		
37	7	3: Joining another person in the garden;<br>3: Interacting with others through non-verbal communications
38	7	3: Joining another person in the garden
39		2: Observing. smelling or touching flowers without prompting
40		2: Observing. smelling or touching flowers without prompting;<br>7
41	5: Repetitive behaviors;<br>7	3: Joining another person in the garden;<br>3: Interacting with others through non-verbal communication
42		2: Observing. smelling or touching flowers without prompting;<br>7
43	5: Repetitive behaviors;<br>7	
44		
45		
46		
47	7	
48		
