# Supplementary material for: Psychological impacts of intervention to improve a therapeutic garden for older adults with dementia: a case study conducted at a care facility
Source: Front Psychiatry. 2023 May 10;14:1183934. doi: 10.3389/fpsyt.2023.1183934 (PMC10206005; doi:10.3389/fpsyt.2023.1183934)
Supplement: Supplementary file 2 [file Table_1.docx]

Psychological impacts of intervention to improve a therapeutic garden for older adults with dementia: A case study conducted at a care facility

**Supplementary material**

**Table S1.** The behaviors mapped.

| **Category** | **Behaviors** |
| --- | --- |
| Passive isolated | Sitting in the garden and snoozing; Sitting in the garden without showing any interest in the garden and its elements; Sitting in the garden in a confusional state; Behaviors focused on the self. |
| Active isolated | Sitting in the garden and observing natural elements or people; Observing, smelling or touching flowers, plants or trees without prompting; Commenting aloud on what the PwD sees, touches or smells; Noticing the presence of animals; Paying attention to outdoor spaces without prompting; Other behaviors (reading, writing, listening to music, smoking, etc.). |
| Social | Talking to other guests, staff members or relatives in the garden; Greeting other guests, staff members or relatives in the garden; Inviting other guests, staff members or relatives to do something together in the garden; Commenting on the garden and its elements with others; Observing, smelling, touching flowers with others. |
| Aggressive | Verbal aggression towards other guests, staff members or relatives; Physical aggression towards other guests, staff members or relatives. |
| Agitated | Laughing awkwardly, crying, shouting; hiding things in the garden; repetitive behaviors; complaining about physical pain, discomfort or state of mind; Sexual behavior. |
| Conscious movement | Going deliberately to a certain part of the garden (alone or with someone). |
| Disoriented movement | Walking, wandering aimlessly |

**Table S2.** PwD (10) whose visits increased versus those who did not visit the garden (or their visits decreased) (11), by demographic variables and baseline measures

|  | **PwD who visited the garden more**  **(N =10)** | **PwD who visited the garden less or not at all**  **(N=11)** |
| --- | --- | --- |
| Age | 79.90 (8.99) | 77.73 (8.37) |
| Education (years of schooling) | 6.30 (2.63) | 7.54 (2.84) |
| MoCA | 13.50 (5.14) | 10.21 (5.61) |
| ADAS-cog | 42.72 (17.23) | 52.24 (20.11) |
| NPI | 16.00 (10.38) | 21.45 (16.63) |
| CDDS | 5.80 (4.42) | 4.73 (4.45) |
| QoL-AD* | 31.10 (3.76) | 27.45 (4.30) |

Note. MoCA: Montreal Cognitive Assessment: ADAS-Cog: Alzheimer’s Disease Assessment Scale -Cognitive subscale; NPI: Neuropsychiatric Inventory; CDDS: Cornell for Depression in Dementia Scale; QoL-AD: Quality of Life - Alzheimer's Disease scale*.* * Kruskal Wallis: z = 1.95 p =.052: tendency of higher scores for the group that visited the garden than for the group that did not.

**Table S3.** Means and standard deviations of the number of visits and behaviors assessed using behavioral mapping before and after the intervention.

| **Behaviors** | **Pre-intervention** | **Post-intervention** |
| --- | --- | --- |
| Passive isolated | 0.10 (0.32) | 1.00 (3.16) |
| Active isolated | 1.80 (1.85) | 4.40 (6.85) |
| Social | 4.30 (3.20) | 9.70 (5.38) |
| Aggressive | 0.00 (0.00) | 0.10 (0.32) |
| Agitated | 0.80 (2.53) | 2.40 (7.59) |
| Conscious movement | 0.40 (1.04) | 0.36 (0.66) |
| Disoriented movement | 1.30 (4.11) | 0.70 (2.21) |

**Table S4**. Mrs. A.’s scores on the measures of interest and her behavioral mapping in the garden before and after the intervention.

|  | **Pre-intervention** | **Post-intervention** |
| --- | --- | --- |
| Measures (score) |  |  |
| MoCA | 10.40 | NA |
| ADAS-cog | 53.30 | NA |
| NPI | 12 | 20 |
| CDDS | 0 | 4 |
| QoL-AD | 30 | 24 |
| Behavioral mapping (frequency) |  |  |
| Visits to the garden | 15 | 22 |
| Passive isolated | 0 | 0 |
| Active isolated | 0 | 14 |
| Social | 3 | 15 |
| Aggressive | 0 | 0 |
| Agitated | 8 | 0 |
| Conscious movement | 0 | 0 |
| Disoriented movement | 13 | 7 |

Note. MoCA: Montreal Cognitive Assessment: ADAS-Cog: Alzheimer’s Disease Assessment Scale -Cognitive subscale; NPI: Neuropsychiatric Inventory; CDDS: Cornell for Depression in Dementia Scale; QoL-AD: Quality of Life - Alzheimer's Disease scale*.*

**Table S5**. Behavioral mapping of Mrs. A. before and after the intervention by observation time (1-48). and behaviors recorded (1. Passive isolated. 2. Active isolated. 3. Social. 4. Aggressive. 5. Agitated. 6. Conscious movement. 7. Disoriented movement). including some specific behaviors noted.

|  | **Behaviors** | |
| --- | --- | --- |
| Behavior observation times 1-48 | **Pre-intervention** | **Post-intervention** |
| 1 |  | 7 |
| 2 |  | 1: Sitting in the garden and observing natural elements or people;  2: Paying attention to outdoor spaces without prompting |
| 3 |  | 2: Paying attention to outdoor spaces without prompting;  2: Observing. smelling or touching flowers. plants or trees without prompting |
| 4 |  | 3: Interacting with others through non-verbal communications |
| 5 |  | 2: Paying attention to outdoor spaces without prompting |
| 6 |  | 3: Giving flowers to staff members;  6: Observing. smelling or touching flowers with other people;  3: Interacting with others through non-verbal communications |
| **7** |  |  |
| 8 |  |  |
| 9 |  |  |
| 10 |  |  |
| 11 |  |  |
| 12 |  |  |
| 13 |  |  |
| 14 |  | 2: Paying attention to outdoor spaces without prompting;  3: Greeting the gardeners; |
| 15 |  | 2: Eating something offered by staff taking a break in the garden;  7 |
| 16 | 3: Greeting the gardeners;  5: Repetitive behaviors;  7 |  |
| 17 | 5: Repetitive behaviors;  7 |  |
| 18 | 7 |  |
| 19 | 5: Repetitive behaviors;  7 |  |
| 20 | 5: Repetitive behaviors; |  |
| 21 |  |  |
| 22 | 5: Repetitive behaviors;  7 |  |
| 23 | 5: Repetitive behaviors;  3: Interacting with others through non-verbal communications |  |
| 24 |  | 7 |
| 25 |  | 3: Interacting with others through non-verbal communications;  3: Joining another person in the garden |
| 26 |  | 1: Sitting in the garden observing natural elements or other people;  2: Noticing the presence of a bird in the garden;  3: Joining another person in the garden |
| 27 |  | 3: Joining another person in the garden |
| 28 | 3: Greeting the gardeners;  7 | 3: Joining another person in the garden;  2: Paying attention to outdoor spaces without prompting |
| 29 | 7 | 2: Paying attention to outdoor spaces without prompting |
| 30 | 7 | 7 |
| 31 |  | 7 |
| 32 |  |  |
| 33 |  |  |
| 34 |  |  |
| 35 |  |  |
| 36 |  |  |
| 37 | 7 | 3: Joining another person in the garden;  3: Interacting with others through non-verbal communications |
| 38 | 7 | 3: Joining another person in the garden |
| 39 |  | 2: Observing. smelling or touching flowers without prompting |
| 40 |  | 2: Observing. smelling or touching flowers without prompting;  7 |
| 41 | 5: Repetitive behaviors;  7 | 3: Joining another person in the garden;  3: Interacting with others through non-verbal communication |
| 42 |  | 2: Observing. smelling or touching flowers without prompting;  7 |
| 43 | 5: Repetitive behaviors;  7 |  |
| 44 |  |  |
| 45 |  |  |
| 46 |  |  |
| 47 | 7 |  |
| 48 |  |  |
